# Supplementary material for: Inhibitory NK receptor expression associates with altered antimalarial function of γδ T cells
Source: PLoS Pathog. 2026 Feb 3;22(2):e1013460. doi: 10.1371/journal.ppat.1013460 (PMC12880742; doi:10.1371/journal.ppat.1013460)
Supplement: S1 Table — (PDF) [file ppat.1013460.s001.pdf]

Fluor	*Marker*	*Clone*	*Supplier*
***Ex vivo* panel**			
Zombie UV	Viability Dye	N/A	Biolegend
BUV395	CD7	M-T701	BD
BUV563	CD16	3G8	BD
BUV737	Vγ9	B3	BD
BV421	KIR3DL1 (CD158e1)	DX9	Biolegend
BV510	CD14	M5E2	Biolegend
BV510	CD19	HIB19	Biolegend
BV711	Pan-γδ TCR	11F2	BD
FITC	Vδ2	123R3	Miltenyi
PE	KIR2DL2/3 (CD158b)	DX27	Biolegend
PE-Dazzle594	LILRB1 (CD85j)	GHI/75	Biolegend
PE-Cy5	CD56 (NCAM)	HCD56	Biolegend
PE-Vio770	NKG2A (CD159a)	REA110	Miltenyi
Alexa647	KIR2DL1 (CD158a)	HP-MA4	Novus
R718	CD3	UCHT1	BD
APC-Cy7	CD28	CD28.2	Biolegend
**Functional panel**			
BV605	IFNγ	4S.B3	Biolegend
BV785	CD107a (LAMP-1)	H4A3	Biolegend
PerCP-Cy5.5	TNFα	MAb11	Biolegend
