# Supplementary material for: Inhibitory NK receptor expression associates with altered antimalarial function of γδ T cells
Source: PLoS Pathog. 2026 Feb 3;22(2):e1013460. doi: 10.1371/journal.ppat.1013460 (PMC12880742; doi:10.1371/journal.ppat.1013460)
Supplement: S1 Fig — Lymphocytes were gated first, followed by single cells, live cells, and then γδ T cells. γδ T cells were gated on CD3+CD14–CD19– cells, followed by pan-γδTCR+, then divided into subsets based on expression of Vγ9 and Vδ2 TCR chains. Representative gating of NKRs on Vγ9+Vδ2+ T cells. KIRs and LILRB1 were gated with FMO samples. Identical gates were applied to all ex vivo phenotyping samples. (PDF) [file ppat.1013460.s002.pdf]

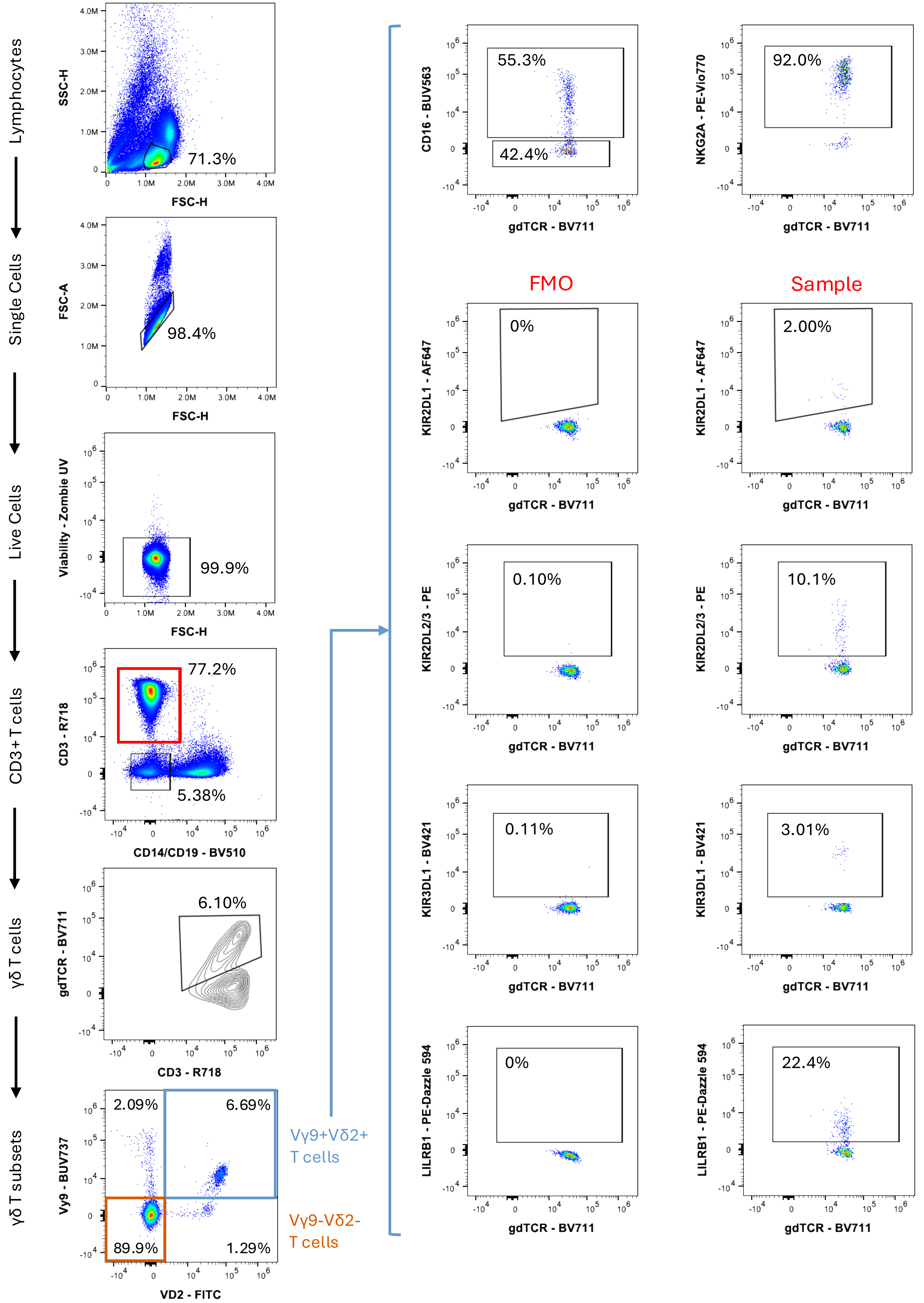

Lymphocytes
Single Cells
Live Cells
CD3+ T cells
γδ T cells
γδ T subsets
SSC-H
FSC-H
71.3%
FSC-A
98.4%
Viability - Zombie UV
99.9%
CD3 - R718
CD14/CD19 - BV510
77.2%
5.38%
gdTCR - BV711
CD3 - R718
6.10%
Vγ9 - BUV737
VD2 - FITC
2.09%
6.69%
89.9%
1.29%
Vγ9+Vδ2+
T cells
Vγ9-Vδ2-
T cells
CD16 - BUV563
55.3%
42.4%
NKG2A - PE-Vio770
92.0%
FMO
Sample
KIR2DL1 - AF647
0%
2.00%
KIR2DL2/3 - PE
0.10%
10.1%
KIR3DL1 - BV421
0.11%
3.01%
LILRB1 - PE-Dazzle 594
0%
22.4%
