# Supplementary figures and images for: Inhibitory NK receptor expression associates with altered antimalarial function of γδ T cells
Source: PLoS Pathog. 2026 Feb 3;22(2):e1013460. doi: 10.1371/journal.ppat.1013460 (PMC12880742; doi:10.1371/journal.ppat.1013460)

**A**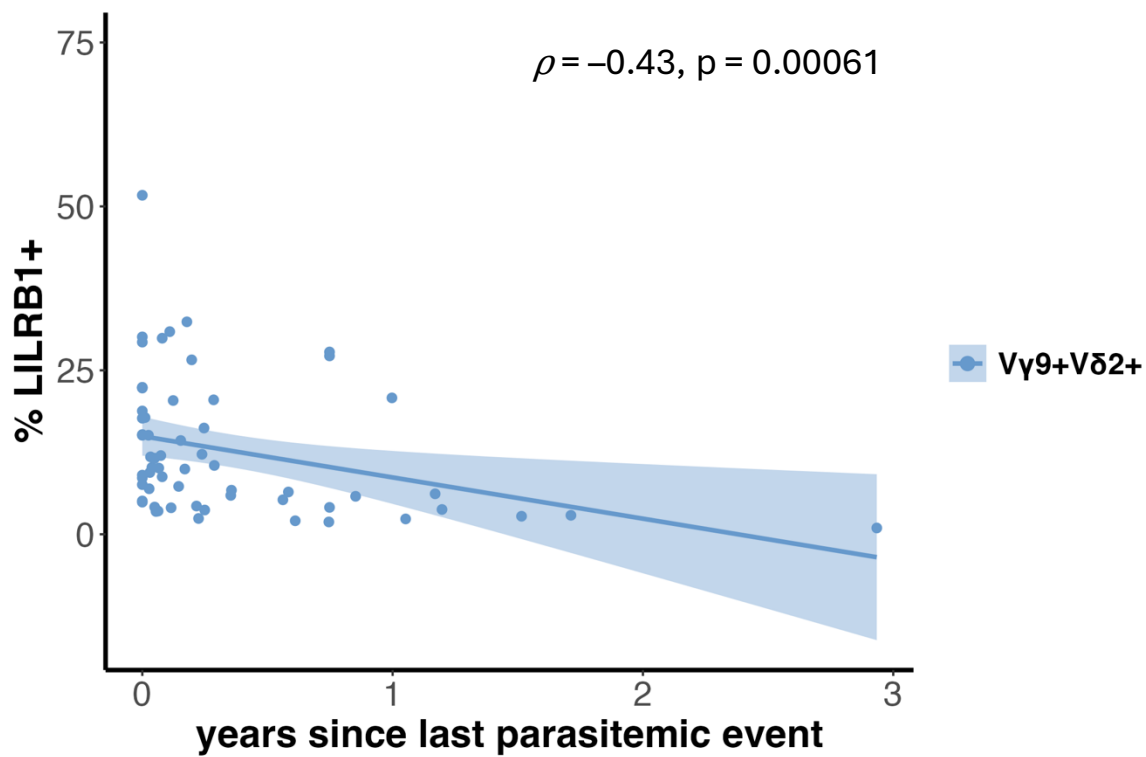**B**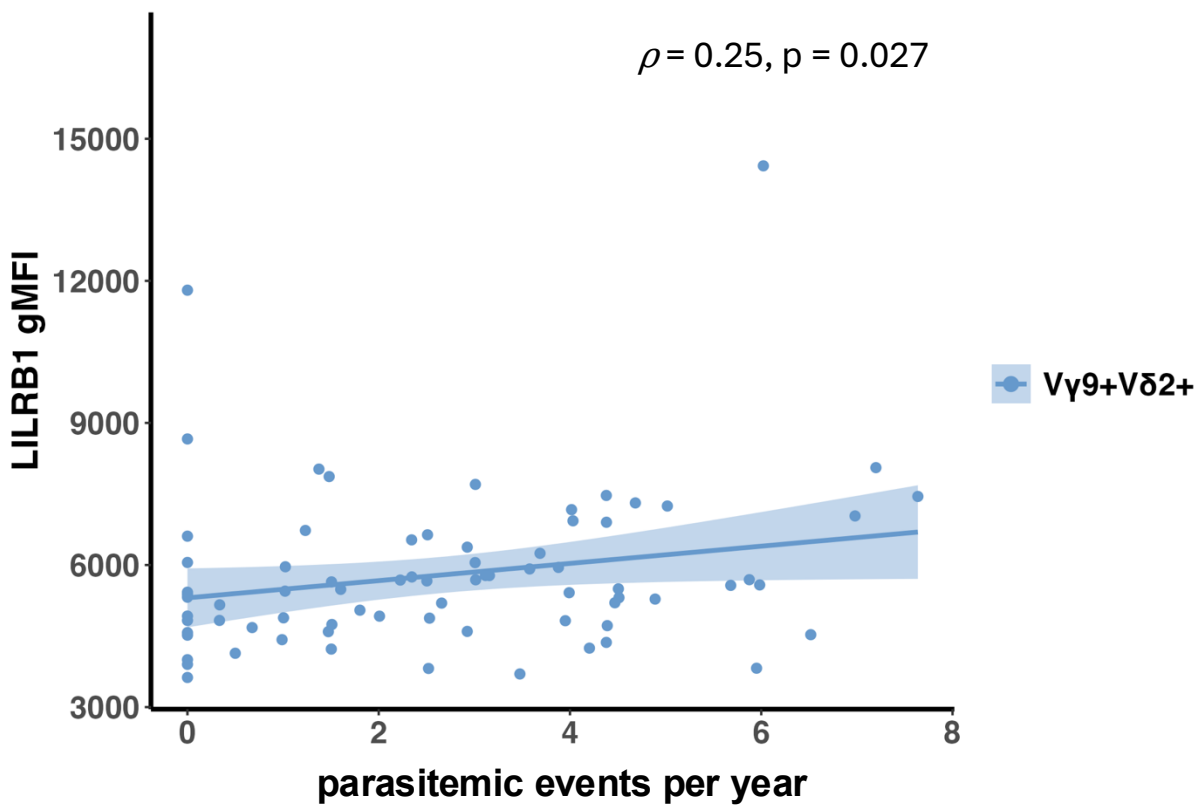

Supplement: S2 Fig — (A) Frequency of LILRB1+ Vγ9+Vδ2+ T cells as a function of time since last parasitemic event. Cases of active parasitemia at the time of sampling were considered t = 0. Individuals with no recorded history of parasitemia were omitted (n = 61). (B) Geometric MFI of LILRB1 on Vγ9+Vδ2+ T cells as a function of parasitemic events per year. ρ = Spearman correlation coefficient. Regression line is a linear best fit with the 95% confidence interval shaded in blue. (PDF) [file ppat.1013460.s003.pdf]

**A**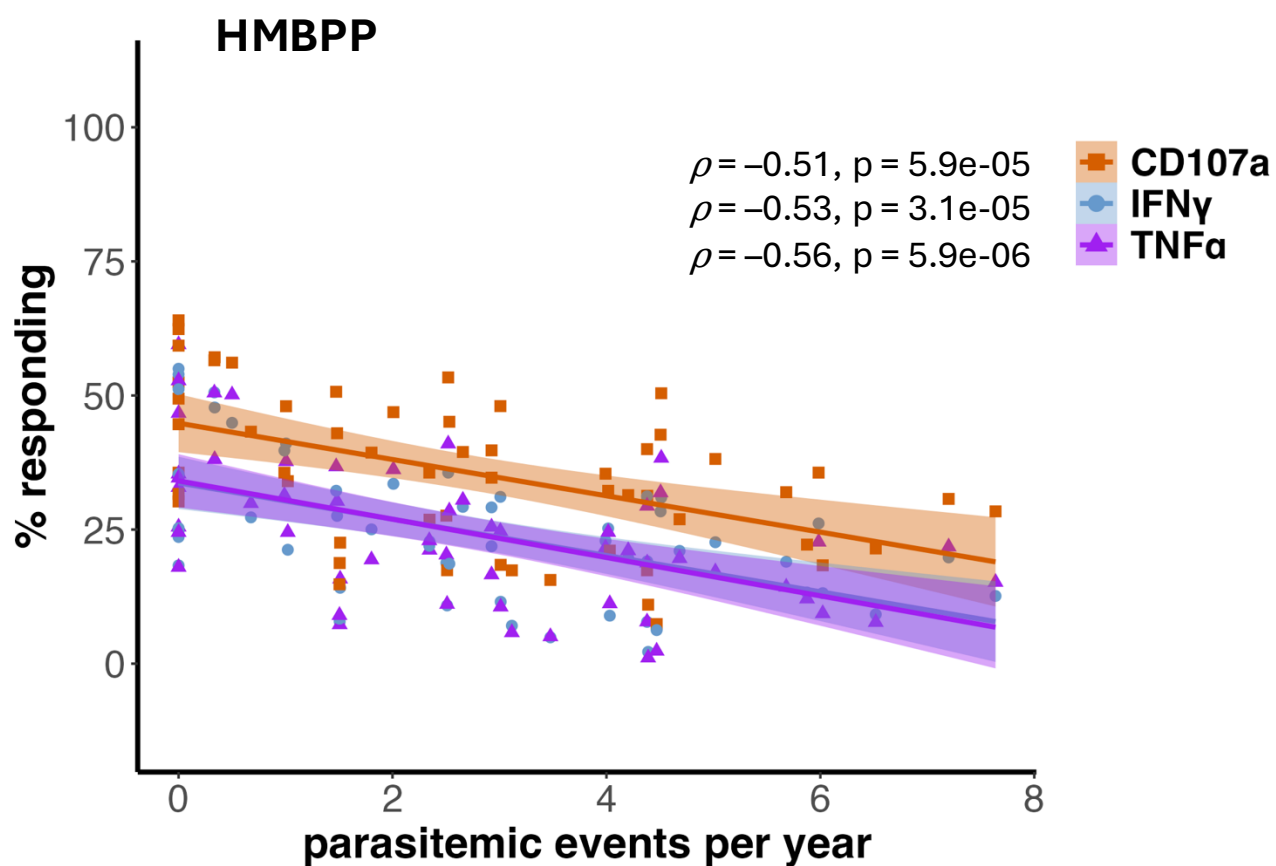**B**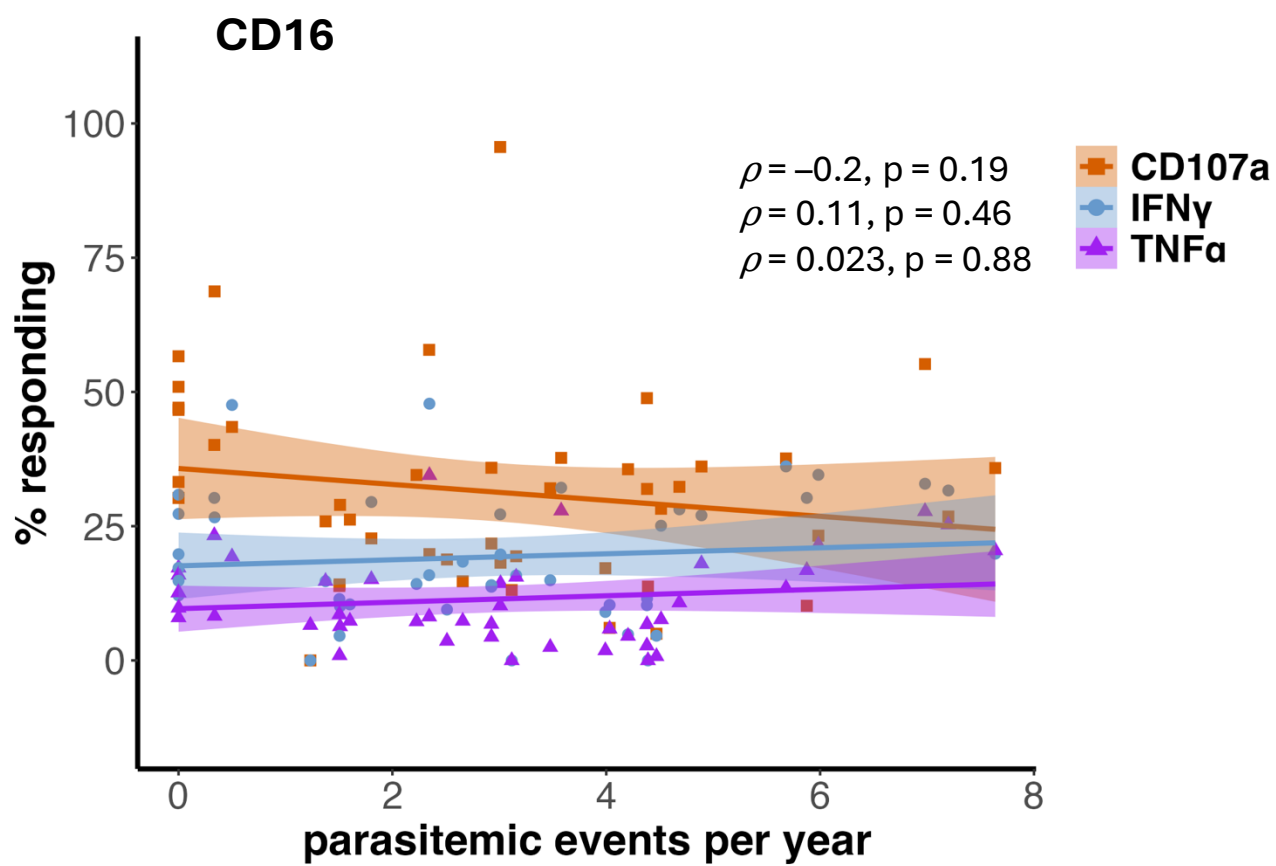

Supplement: S4 Fig — Functional response of total Vγ9+Vδ2+ T cells as a function of parasitemic events per year, measured by surface mobilization of CD107a and production of IFNγ and TNFα upon a 5-hour stimulation of PBMC with (A) HMBPP (n = 56) or (B) plate-bound α-CD16 crosslinking antibody (n = 45). All data is background subtracted. ρ = Spearman correlation coefficient. Regression lines are a linear best fit with the 95% confidence interval shaded in orange (CD107a), blue (IFNγ), or purple (TNFα). (PDF) [file ppat.1013460.s005.pdf]

**A**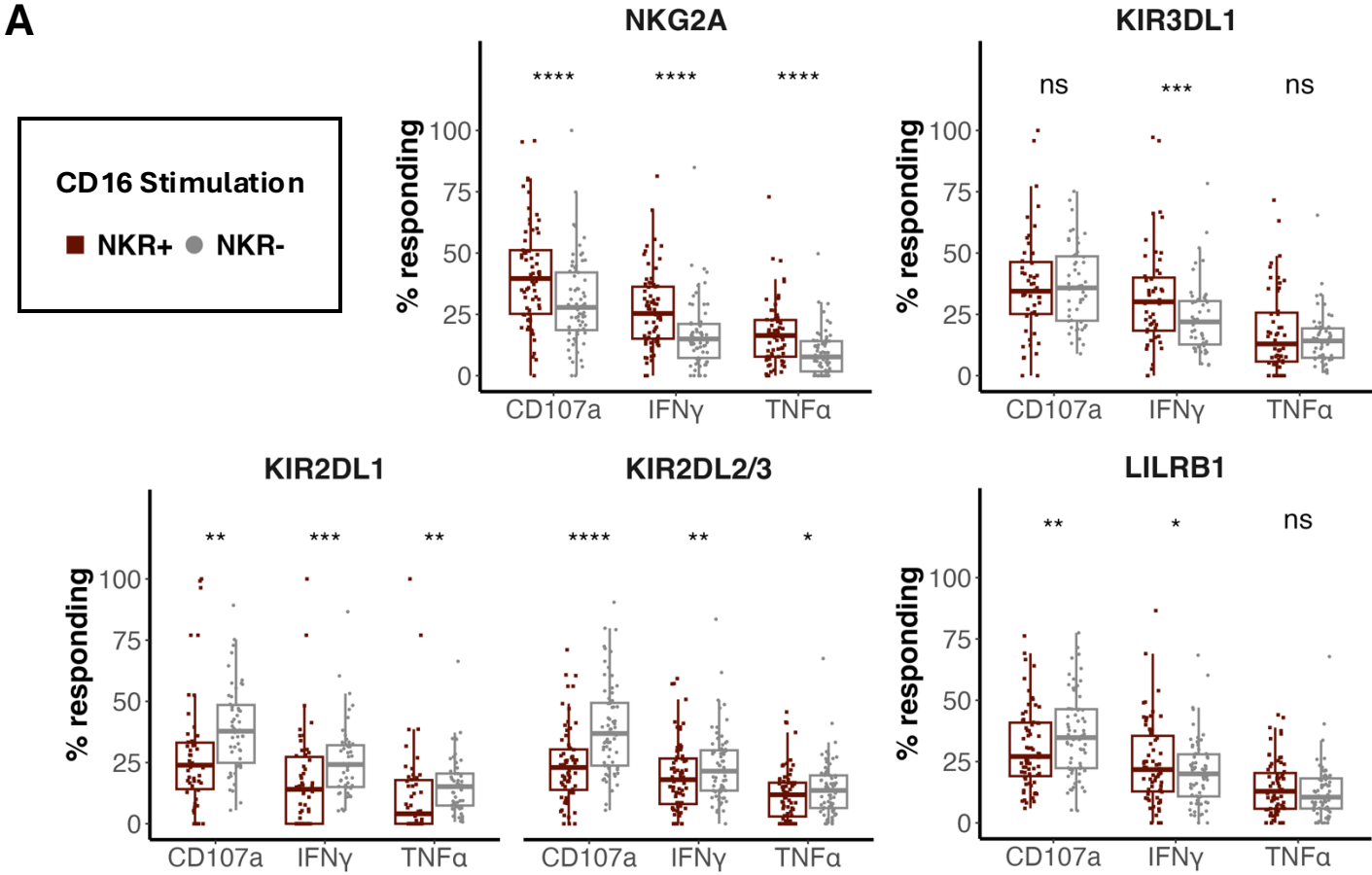**B**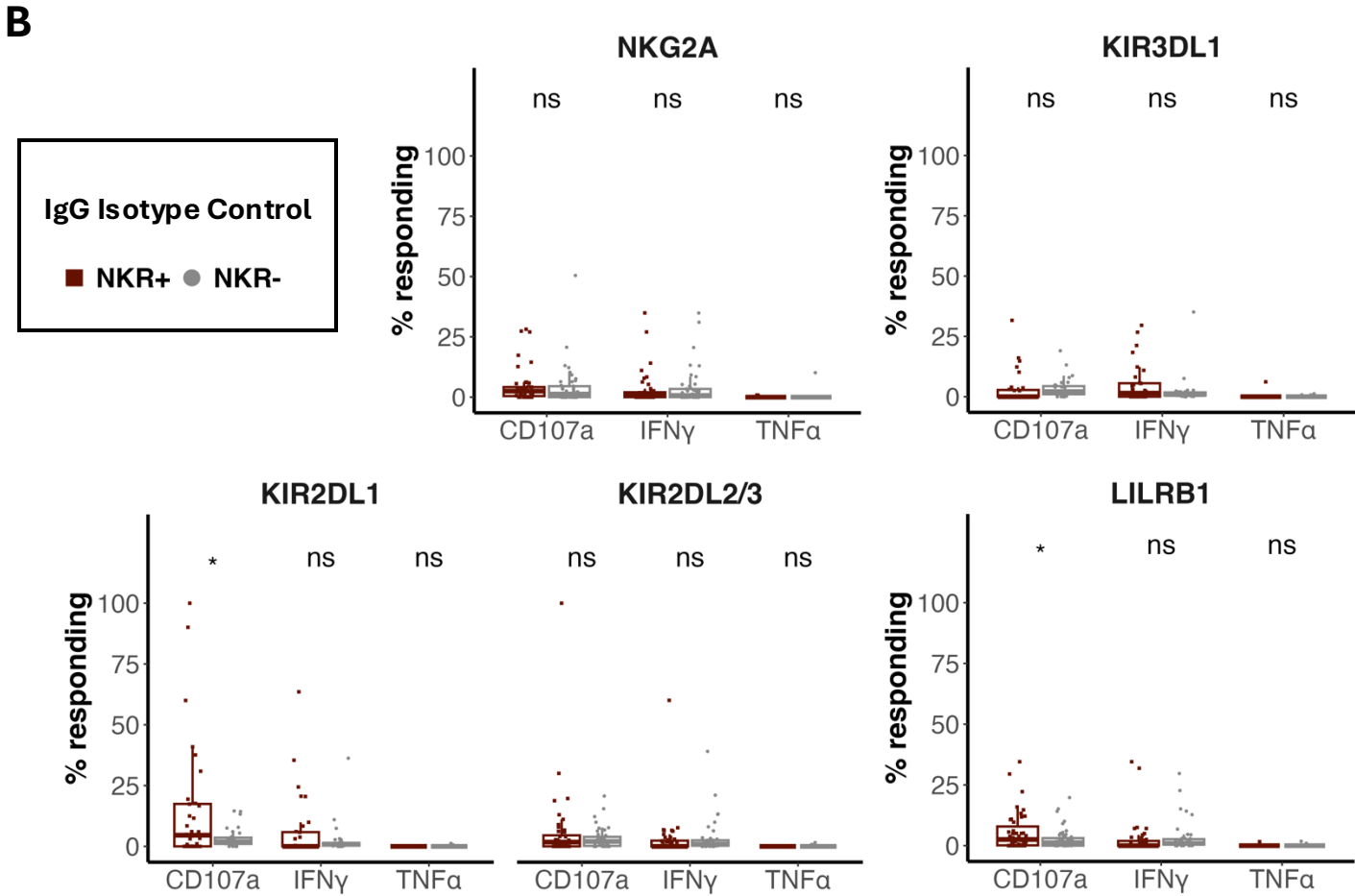

Supplement: S5 Fig — (A) Functional response of NKR+ vs. NKR– Vγ9+Vδ2+ T cells, as measured by surface mobilization of CD107a and production of IFNγ and TNFα upon a 5-hour stimulation with plate-bound α-CD16 crosslinking antibody. Data is not background subtracted. NKG2A: n = 69; KIR3DL1: n = 51; KIR2DL1: n = 50; KIR2DL2/3: n = 68; LILRB1: n = 67. (B) Background response of NKR+ vs. NKR– Vγ9+Vδ2+ T cells stimulated with plate-bound isotype control IgG1 antibody. NKG2A: n = 45; KIR3DL1: n = 31; KIR2DL1: n = 32; KIR2DL2/3: n = 44; LILRB1: n = 43. NKR expression categories were compared via paired Wilcoxon signed rank test, with datapoints paired by donor. For all analyses, a 2-tailed P value < 0.05 was considered significant (*: p <= 0.05, **: p <= 0.01, ***: p <= 0.001, ****: p <= 0.0001). Data were included if parent gate contained a minimum of 10 cells. (PDF) [file ppat.1013460.s006.pdf]

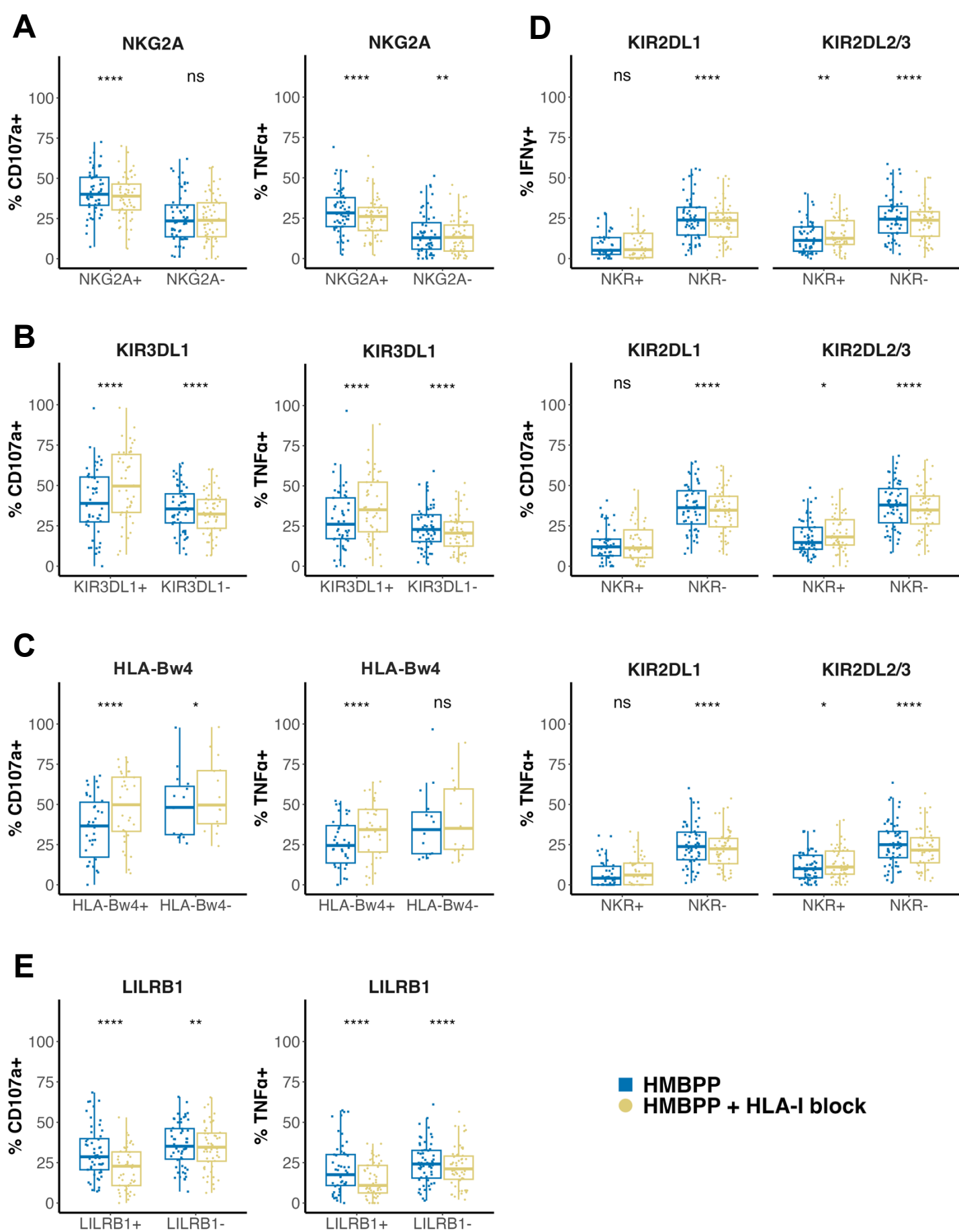

Supplement: S6 Fig — (A) Frequency of CD107a+ (left) and TNFα+ (right) NKG2A+ or NKG2A– Vγ9+Vδ2+ T cells stimulated with HMBPP vs. HMBPP + HLA-I block (NKG2A+: n = 56; NKG2A–: n = 56). (B) Frequency of CD107a+ (left) and TNFα+ (right) KIR3DL1+ or KIR3DL1– Vγ9+Vδ2+ T cells stimulated with HMBPP vs. HMBPP + HLA-I block (KIR3DL1+: n = 49; KIR3DL1–: n = 56). (C) Frequency of CD107a+ (left) and TNFα+ (right) KIR3DL1+ Vγ9+Vδ2+ T cells grouped by individuals with (n = 34) or without (n = 15) the HLA-Bw4 allele in HMBPP vs. HMBPP + HLA-I block conditions. (D) Frequency of IFNγ+ (top), CD107a+ (middle) and TNFα+ (bottom) KIR+ or KIR– Vγ9+Vδ2+ T cells stimulated with HMBPP vs. HMBPP + HLA-I block (KIR2DL1+, n = 42; KIR2DL1–, n = 56; KIR2DL2/3+, n = 54; KIR2DL2/3–, n = 56). (E) Frequency of CD107a+ (left) and TNFα+ (right) LILRB1+ or LILRB1– Vγ9+Vδ2+ T cells stimulated with HMBPP vs. HMBPP + HLA-I block (LILRB1+: n = 52; LILRB1–: n = 56). All data were background-subtracted with matched unstimulated samples, and groups were compared with a paired Wilcoxon signed rank test. For all analyses, a 2-tailed P value < 0.05 was considered significant (*: p <= 0.05, **: p <= 0.01, ****: p <= 0.0001). Data were included if parent gate contained a minimum of 10 cells. (PDF) [file ppat.1013460.s007.pdf]
