# Supplementary material for: Inhibitory NK receptor expression associates with altered antimalarial function of γδ T cells
Source: PLoS Pathog. 2026 Feb 3;22(2):e1013460. doi: 10.1371/journal.ppat.1013460 (PMC12880742; doi:10.1371/journal.ppat.1013460)
Supplement: S3 Fig — Lymphocytes were gated first, followed by single cells, live cells, and then γδ T cells. γδ T cells were gated on CD3+CD14–CD19– cells, followed by pan-γδTCR+, then divided into subsets based on expression of Vγ9 and Vδ2 TCR chains. Representative gating of response markers on Vγ9+Vδ2+ T cells. CD107a, IFNγ, and TNFα were gated with corresponding unstimulated/isotype control samples. Identical gates were applied to all HMBPP-, HMBPP + HLA-I block-, and CD16-stimulated samples. (PDF) [file ppat.1013460.s004.pdf]

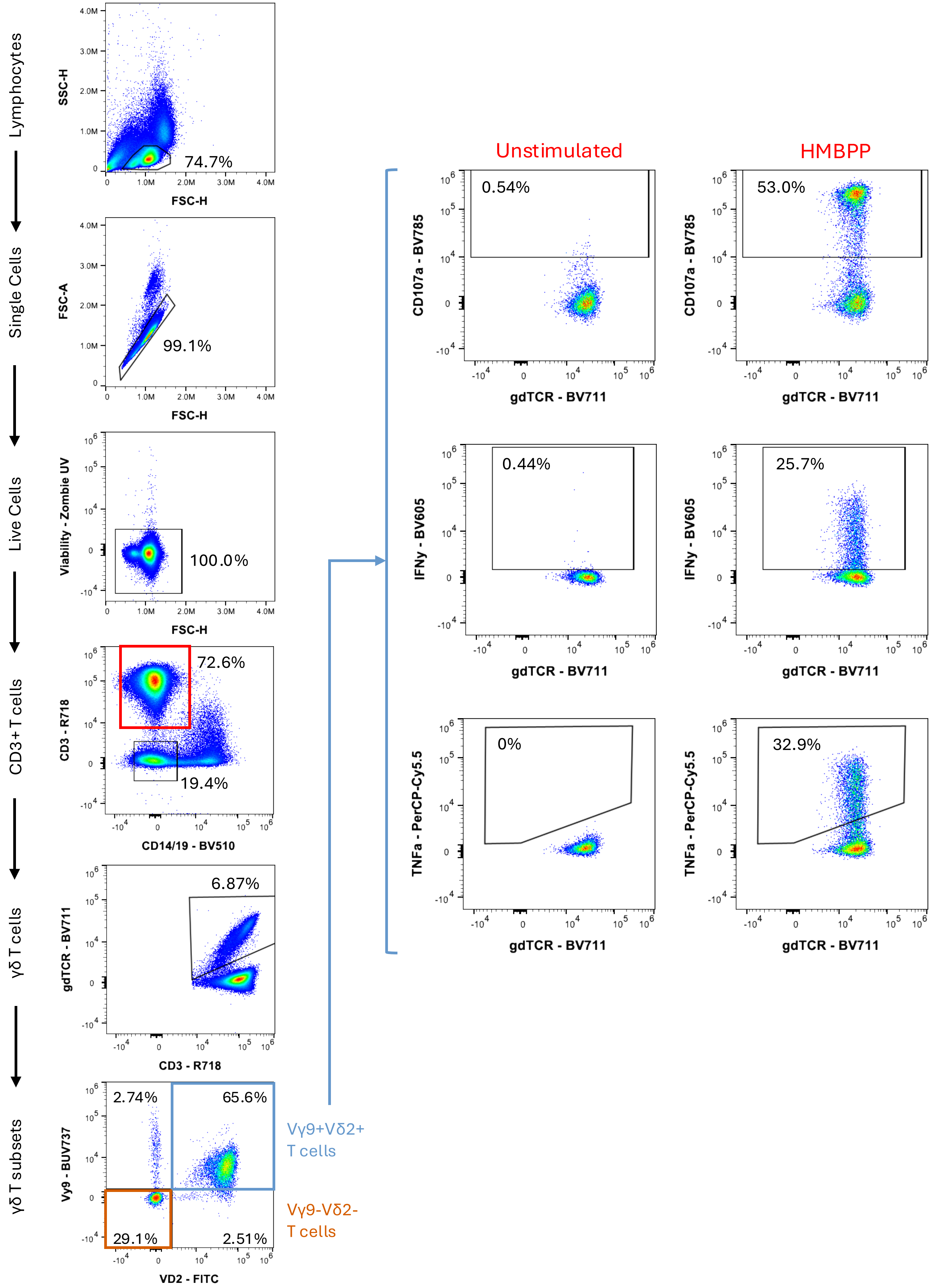

Lymphocytes
Single Cells
Live Cells
CD3+ T cells
γδ T cells
γδ T subsets
SSC-H
FSC-H
74.7%
FSC-A
99.1%
Viability - Zombie UV
100.0%
CD3 - R718
CD14/19 - BV510
72.6%
19.4%
gdTCR - BV711
CD3 - R718
6.87%
Vγ9 - BUV737
VD2 - FITC
2.74%
65.6%
29.1%
2.51%
Vγ9+Vδ2+
T cells
Vγ9-Vδ2-
T cells
Unstimulated
HMBPP
CD107a - BV785
0.54%
53.0%
IFNγ - BV605
0.44%
25.7%
TNFa - PerCP-Cy5.5
0%
32.9%
gdTCR - BV711
